# Resolution of Gastroesophageal Reflux Disease Following Correction for Upper Cross Syndrome—A Case Study and Brief Review

**DOI:** 10.3390/clinpract11020045

**Published:** 2021-05-21

**Authors:** Eric Chun-Pu Chu, Kenneth R. Butler

**Affiliations:** 1 Department of Chiropractic and Physiotherapy, New York Medical Group, 41F 8 Argyle Street, Mong Kok, Hong Kong; 2 School of Medicine, University of Mississippi Medical Center, 2500 N State, Jackson, MS 39216, USA; kbutler@umc.edu

**Keywords:** chiropractic manipulation, forward head, forward shoulder posture, gastroesophageal reflux disease, hyperkyphosis, upper cross syndrome

## Abstract

Upper cross syndrome (UCS) is a condition caused from prolonged poor posture manifesting as thoracic hyperkyphosis with forward head and shoulder postures. It has been associated with several other secondary conditions, causing pain and discomfort to those with the condition. This is a case report of a 35-year-old female presenting to clinic with a sharp pain in the neck, upper back, and sternum area for 4 weeks and gastroesophageal reflux disease (GERD). She had been working at home for several months after the shelter at home order was issued. Following evaluation and corrective treatment with cervical adjustment and soft tissue massage, the patient’s posture improved and reported full pain resolution. Her symptoms of GERD concurrently resolved as well. She continued to receive chiropractic adjustment two times per month for correcting spinal misalignment. Full restoration of posture was attained on the full spine radiographs at 9 months follow-up. The patient remained symptom-free at 12 months follow-up. Manipulative and preventive therapies aimed at treating and preventing UCS should be more widely adopted to prevent secondary conditions.

## 1. Introduction

Upper cross syndrome (UCS) is a common postural problem among the young and old and is described as thoracic hyperkyphosis with forward head and shoulder postures [1]. Usually, the postural changes resulting in UCS are occupationally induced in persons required to assume static postures for prolonged periods of time [1,2]. In UCS, muscles including the serratus and lower trapezius become weak resulting in a tightening of the pectoral and neck muscles [3]. Many patients present with shoulders that have become more rounded and hunched with the neck and head craning forward [4,5]. The spine will also curve inward near the neck and outward in the upper back and shoulder area [4,5]. Commonly reported symptoms include headaches, neck pain or strain in the back of the neck, chest pain and tightness, upper back pain, restricted range of motion in the neck or shoulders, and numbness and tingling in upper arms [6,7,8]. Other patients report difficulty with various activities, such as sitting, reading, or driving for long periods of time [9].

## 2. Case Report

A 35-year-old female graphic designer presented with sharp pain in the neck, upper back, and sternum area of 4 weeks duration. The pain radiated to substernal, interscapular, and subscapular areas as a gripping and squeezing sensation without numbness. The symptoms were worse in the evening, and lying on her back while sleeping aggravated the pain. Her medical history indicated a chronic history of neck pain, heart burn, acid reflux, and period pain. After the lock-down during the pandemic, she remained inside her apartment and reported sitting on the bed at her computer while working. Her neck pain gradually became severe and was rated at 8/10 on a numeric pain scale. Her laboratory, barium swallow study and orthopedic tests were negative and ruled out major visceral pathology. The patient reported supragastric belching and regurgitation for at least six months and sometimes experienced a burning sensation in the chest. Changing position from sitting to standing could partially relieve acid reflux. She denies dysphagia, vomiting or blood in her stool. Upper gastrointestinal endoscopy performed two months prior revealed mild inflammation in the distal esophagus and at the gastroesophageal junction, compatible with gastroesophageal reflux disease (GERD). She was prescribed antacids, histamine 2 receptor blockers, and dietary restriction which provided temporary relief only for the heartburn. The patient then sought chiropractic care for her neck pain.

**Figure 1:**
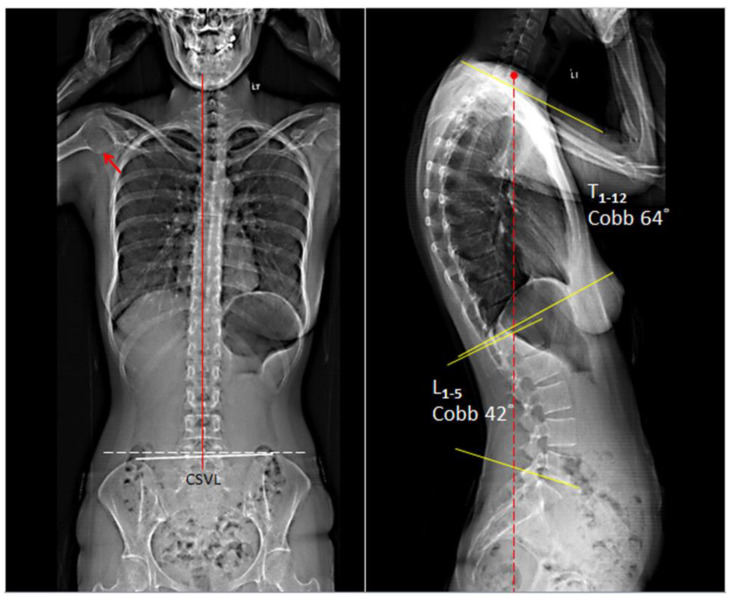
EOS^®^ radiographs demonstrating degenerative spondylosis of the lower cervical spine with osteophytes at posterior C5 and C6, C7/T1 disc-space narrowing, straightened cervical lordosis and thoracic hyperkyphosis, and cystic change of the right humeral head (red arrow). In general, the average angle of thoracic kyphosis and lumbar lordosis is 43.55° ± 6.44 and 32.42° ± 6.29, respectively. CSVL: central sacral vertical line (red line).

Upon initial evaluation, the patient presented with a forward head and slouching posture. Joint mobility was assessed and indicated restriction at C5–7 and T10-L2 levels. Muscular hypertonicity was evaluated and indicated present in bilateral upper trapezius, levator scapulae, sternocleidomastoid, and subscapularis. Her cervical range of motion was limited and painful at 10° extension (normal > 60°) and 45° of right rotation (normal > 80°). The orthopedic and neurological examinations were within normal limits. Full spine EOS^®^ radiographs showed a picture of degenerative spondylosis of the lower cervical spine with osteophytes at posterior C5 and C6, C7/T1 disc-space narrowing, straightened cervical lordosis, thoracic hyperkyphosis, induced lumbar kyphosis, and cystic change of the right humeral head (Figure 1). Patients presented with a global thoracic curve of 64° and lumbar curve of 42°. In general, the average angle of thoracic kyphosis and lumbar lordosis is 43.55 ± 6.44 and 32.42 ± 6.29, respectively. Based on the symptoms of heartburn relating with neck pain and the exclusion of systemic disorders, the patient was given a diagnosis of upper cross syndrome with GERD.

**Figure 2:**
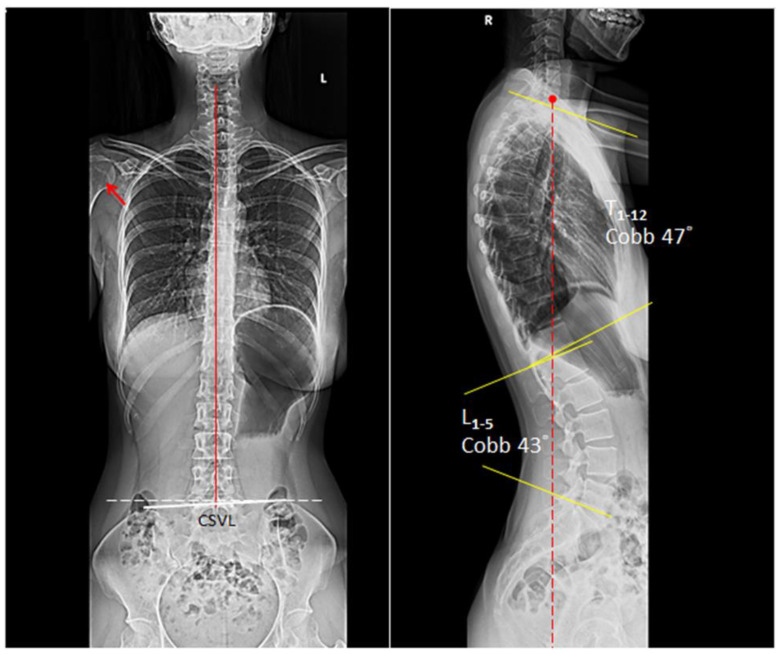
At 9 months follow-up, the thoracic kyphotic angle reduced to 47° and the GERD also resolved. The patient remained pain- and GERD-free at the 12-month follow-up visit. In a balanced state, the C7 plumb line (dashed red line) should fall within 3 cm, either anterior or posterior, of the posterosuperior corner of the S1 endplate.

The patient was treated with cervical adjustment and soft tissue massage (STRIG Massage Device, Seoul, Korea) three times per week with emphasis on restoring mobility to stiff joints and relieving muscle tightness [10,11,12]. At the end of first month, the patient reported that her neck and upper back pain was fully recovered. She also stopped all medications as her symptoms of heartburn had concurrently eliminated. A rehabilitation program including tuck chin exercises and lifestyle modification was added to the second phase of treatment plan to strengthen the muscles [7,13,14,15]. The patient continued to receive chiropractic adjustment twice per month for correcting spinal alignment. Restoration of the posture was demonstrable on the full spine radiographs at 9 months follow-up (Figure 2). At 12 months after initiating treatment, the patient remained healthy, free of pain and GERD, in the pandemic home-office.

## 3. Discussion

Upper cross syndrome is characterized anteriorly by weakened deep neck flexor muscles and tight pectoralis and sternocleidomastoid muscles and posteriorly by thigh upper trapezius and levator scapulae and weak rhomboids, lower trapezius, and the serratus anterior muscles resulting from poor posture [1]. Though already common among office workers with poorly designed ergonomic workstations, there has been an increased prevalence because of the increased number of people working from home during the COVID-19 lockdown global pandemic. The pandemic, as well as UCS, has brought about additional non-viral/non-infectious health conditions affecting population health and function. Spending numerous hours sitting at a computer, watching television or being on a smartphone often contributes to the type of poor posture and dysfunctional structural adaptation associated with UCS. UCS is known to contribute or exacerbate a myriad of symptoms related to other concomitant conditions [6,16,17,18]. Slouched posture from working on a laptop without ergonomic office equipment, such as an appropriate desk and chair, can lead to UCS and previously unreported consequences such as the development of GERD.

UCS has been associated with other pathological conditions including decreased respiratory function, cervicogenic headache, and increased risk of fractures [2,17,18]. In this case, UCS was the likely culprit triggering symptoms of heartburn caused by acid reflux. Physiologically, working in a poor postural position for extended periods of time places pressure on the abdomen and can force acidic stomach contents through the lower esophageal sphincter causing significant discomfort.

As demonstrated in this case and with most musculoskeletal conditions, the best way to fight UCS is through preventative exercises and postural improvement [11,12,13,14,15]. Once UCS has progressed, treatment approaches include procedures aimed at strengthening the weakened posterior musculature and the stretching of the tightened anterior musculature [19]. The cervical adjustment and soft tissue massage techniques are synergistic procedures that were very effective in a quick resolution of UCS with concurrent resolution of GERD in this patient. Ultimately, correction of the anatomical deviations resulting from UCS also led to cessation of pharmacologic treatment of GERD. The positive outcomes in this case resulted from a combination of therapies used to loosen tight muscles and tighten the weaker muscles as appropriate and allowing the anatomy to return to a more normal, homeostatic position. Patient compliance and adherence to treatment regimens were also responsible for the final resolution of this condition. Continuing exercise plans and maintaining good postural habits over the life course will likely prevent future reoccurrence.

## 4. Conclusions

Presented is a case of upper cross syndrome with coexisting GERD. GERD is secondary to the musculoskeletal changes resulting from prolonged periods of working from home during the peak of the COVID-19 pandemic. While the musculoskeletal abnormalities and GERD were corrected with appropriate therapy, this case demonstrates that a sedentary lifestyle plays a significant role in the population being more susceptible to additional pathological conditions when posture is compromised from repetitive and excessive sitting.

## Data Availability

Not applicable.
